# Altitude influences microbial diversity and herbage fermentation in the rumen of yaks

**DOI:** 10.1186/s12866-020-02054-5

**Published:** 2020-12-04

**Authors:** Qingshan Fan, Metha Wanapat, Tianhai Yan, Fujiang Hou

**Affiliations:** 1 State Key Laboratory of Grassland Agro-ecosystems, Key Laboratory of Grassland Livestock Industry Innovation, Ministry of Agriculture, College of Pastoral Agriculture Science and Technology, Lanzhou University, Lanzhou, 730000 China; 2 Tropical Feed Resources Research and Development Center (TROFREC), Department of Animal Science, Faculty of Agriculture, Khon Kaen University, Khon Kaen, 40002 Thailand; 3 Agri-Food and Biosciences Institute, Hillsborough, County Down BT26 6DR UK

**Keywords:** Yak, Rumen microbiota, VFA profiles, Fermenting ability, High altitude

## Abstract

**Background:**

Rumen microbiota in ruminants are vital for sustaining good rumen ecology, health, and productivity. Currently, limited information is available regarding the response of yaks (*Bos grunniens*) to fluctuating environments, especially the rumen microbiome. To address this, we investigated the diet, rumen bacterial community, and volatile fatty acids (VFA) of rumen fluid of yaks raised in the great Qinghai-Tibet plateau (QTP) at 2800 (low altitude, L), 3700 (middle altitude, M), and 4700 m (high altitude, H) above sea level.

**Results:**

The results showed that despite a partial diet overlap, H yaks harbored higher fibrous fractious contents than the M and L grazing yaks. Bacteria including *Christensenellaceae_R-7_group*, *Ruminococcus_1*, *Romboutsia*, *Alloprevotella*, *Eubacterium coprostanoligenes*, *Clostridium*, *Streptococcus*, and *Treponema* were found to be enriched in the rumen of yaks grazing at H. They also showed higher rumen microbial diversity and total VFA concentrations than those shown by yaks at M and L. Principal coordinates analysis (PCoA) on weighted UniFrac distances revealed that the bacterial community structure of rumen differed between the three altitudes. Moreover, Tax4fun metagenome estimation revealed that microbial genes associated with energy requirement and carbohydrate metabolic fate were overexpressed in the rumen microbiota of H yaks.

**Conclusions:**

Collectively, our results revealed that H yaks had a stronger herbage fermenting ability via rumen microbial fermentation. Their enhanced ability of utilizing herbage may be partly owing to a microbiota adaptation for more energy requirements in the harsh H environment, such as lower temperature and the risk of hypoxia.

**Supplementary Information:**

The online version contains supplementary material available at 10.1186/s12866-020-02054-5.

## Background

The rumen is a complicated microbial ecosystem harboring compartment, hosting abundant bacteria, protozoa, and fungi, that play vital roles in ruminants [1]. Ruminants depend on their rumen microbes for degradation of structural carbohydrates (cellulose, hemicellulose, and lignin) in herbage, and synthesis of rumen volatile fatty acids (VFA) and microbial proteins synthesis as energy and protein sources [2]. A previous study suggested that the rumen is considered to be free of microbes after birth, but is soon contaminated with microbes from the dam and surrounding environment [3]. The rumen microbial consortium provides useful functions for the host, such as food fermentation [4], immunity regulation [5], disease preventive measures [6], energy balance [7], and physical development [8]. Some experiments have shown that fluctuation of the rumen microbial consortium can lead to a shift in its function [4, 9]; nevertheless, it is still unknown how rumen microbiota composition and function influence response to various environmental factors.

The Qinghai-Tibet Plateau (QTP), located in the southwest part of China and known as the Earth’s third pole, is the highest and largest plateau on the planet. The major land use on this plateau has been for grazing livestock since ancient times. It has been reported that more than 15 million yaks (*Bos grunniens*) are raised on the QTP, accounting for approximately 90% of the total yak population worldwide [10]. Yaks are one of the world’s most treasured domesticated livestock. They are an iconic symbol of Tibet and high elevation because of their ability to thrive in an extremely harsh living environment [2]. Under traditional management practices, yaks commonly graze in a full-grazing system with herbage as the only feed [4]. Yaks play an essential function in the alpine ecosystem in various ways, including enhancing plant diversity through creation of micro-habitats, providing a livelihood for local herdsmen, enhancing soil structure, and promoting the material circulation and energy flow in the ecosystem [10]. These unique characteristics result in an attractive system explore the adaptation process of yaks to altitudinal gradients. First, because the main characteristics of high altitude areas are low pressure, low oxygen, low temperature, high sunlight, and climate dynamics [11], the ecological mechanisms associated with rumen microbiota consortium could respond in a different manner compared with those of low altitude yaks. Second, yaks have adapted well to high altitude environments through long-term evolution [9]. However, the adjustment of microbial communities to high altitude areas is still difficult to explain, as the rumen flora is related to nutrition and metabolism of the host. Finally, yaks are ruminants and their rumen are usually considered free of microbes at birth; hence, the rumen microorganisms of calves would be obtained from the environment and mother’s milk [2]. Therefore, the composition of yak rumen flora is affected by both the host and the environmental conditions. This makes investigations more complicated compared to non-host environmental microbial communities.

Previous studies have studied the relationship between yak rumen microbiota and host factors (e.g., age, health status, rumen region) and the environment (e.g., diet, environmental microbes) [2, 12–14]. High-altitude grazing animals, such as Tibetan sheep and yak, harbor similar rumen microbiota but different microbial interactions when compared with their low-altitude relatives [9]. There is little data on how high-altitude yaks acclimate to harsh conditions, such as cold climate and hypoxic environments, from the perspective of rumen microbiota. Therefore, we explored how the rumen microbiota responds to the adaptation process of yaks to high-altitude environments. In this study, we investigated the yak diet profile, rumen microbiota composition, and VFA profiles at different altitudes. We addressed three critical questions: (1) Do the yaks have different rumen microbiota diversity and fermentation ability (VFA profiles) at different elevations? (2) Is there a link between rumen microbiota and VFAs? (3) Does rumen microbiota composition and function covary at and due to different elevations? Our results are significant for studying microbiota adaptation to higher energy demands of the associated hosts under harsh conditions such as cold climate and hypoxic high-altitude environmental conditions.

## Results

### Chemical composition of herbage samples

The nutrient composition of mixed edible herbage samples collected from the three altitudes of the QTP is presented in Table S1. The neutral detergent fiber (NDF) content increased with altitude (*P <* 0.05). However, the acid detergent fiber (ADF) content was the highest (*P <* 0.05) in H and the lowest in M (*P <* 0.05). There were no significant differences in the contents of dry matter (DM), ether extract (EE), crude protein (CP), and organic matter (OM) (*P >* 0.05) in herbage from the three altitudes.

### Rumen fermentation parameters

There were no significant differences in yak rumen pH, NH_3_-N, isobutyrate, valerate, and isovalerate concentrations between the three altitudes (*P* > 0.05; Table 1), whereas, the rumen total VFA (TVFA) concentration increased with altitude (*P <* 0.05). The proportions of acetate and propionate in H were higher than in M and L (*P <* 0.05). The proportion of butyrate at low and middle altitudes was higher than at high altitude (*P <* 0.05).

**Table 1:** Effects of herbage from the three elevations on ruminal fermentation in yaks

Item	Altitude ^d^			SEM ^e^	*P* value
	L	M	H		
pH	6.76	6.73	6.69	0.0234	0.4585
NH_3_-N (mg/L)	79.43	75.14	73.04	1.5017	0.2125
TVFA (mmol/L)	21.31^c^	37.10^b^	40.64^a^	1.5587	0.0026
Acetate (%)	72.53^b^	73.32^b^	77.88^a^	0.5994	0.0060
Propionate (%)	11.53^b^	12.14^b^	13.78^a^	0.3456	0.0179
Butyrate (%)	11.62^a^	11.87^a^	4.84^b^	0.6264	0.0013
Isobutyrate (%)	1.45	1.47	1.51	0.0525	0.9170
Valerate (%)	1.26	1.14	1.13	0.0482	0.5054
Isovalerate (%)	0.81	0.83	0.85	0.0343	0.9101

^a,b,c^ Values in the same row with different superscript letters differ significantly (*P* < 0.05)

^d^ L, 2800 m; M, 3700 m; H, 4700 m

^e^ Standard error of the mean

### Composition of bacterial population found in rumen fluid

**Fig. 1:**
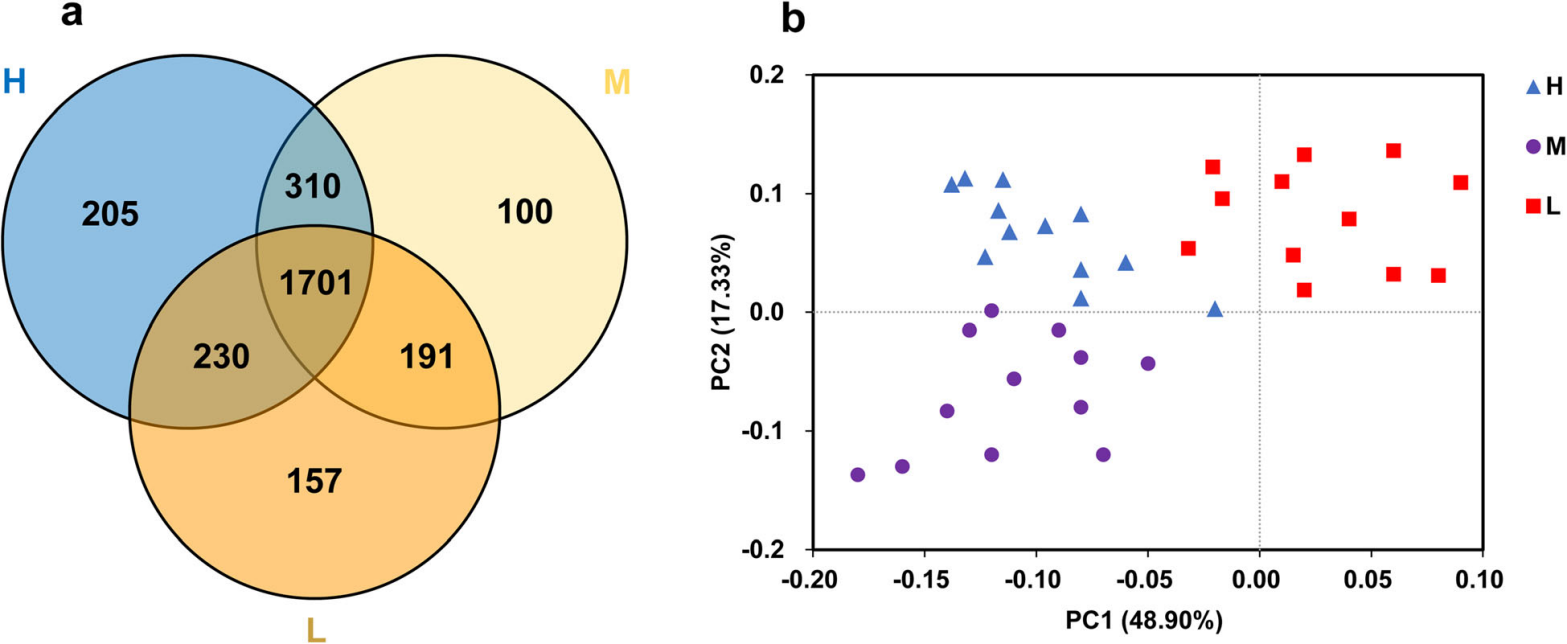
Differences in bacterial community richness and OTUs at different elevations. Venn diagram (**a**) showing the different and similar OTUs at the three elevations. Principal coordinate analysis (PCoA) (**b**) of the yak ruminal microbiota at the three elevations. The PCoA plots were constructed using the weighted Unifrac method. L, 2800 m; M, 3700 m; H, 4700 m

**Fig. 2:**
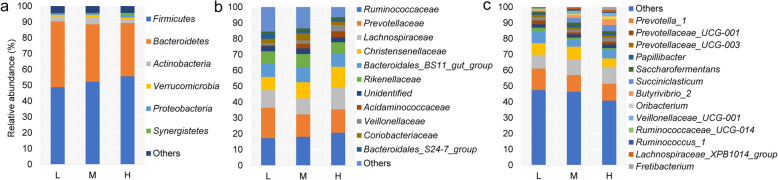
Bacterial comparisons of the rumen in yaks grazing pastures at the three elevations (L, 2800 m; M, 3700 m; H, 4700 m). The relative abundance of (**a**) bacterial phyla, (**b**) family, and (**c**) genus were obtained to be > 1%

**Fig. 3:**
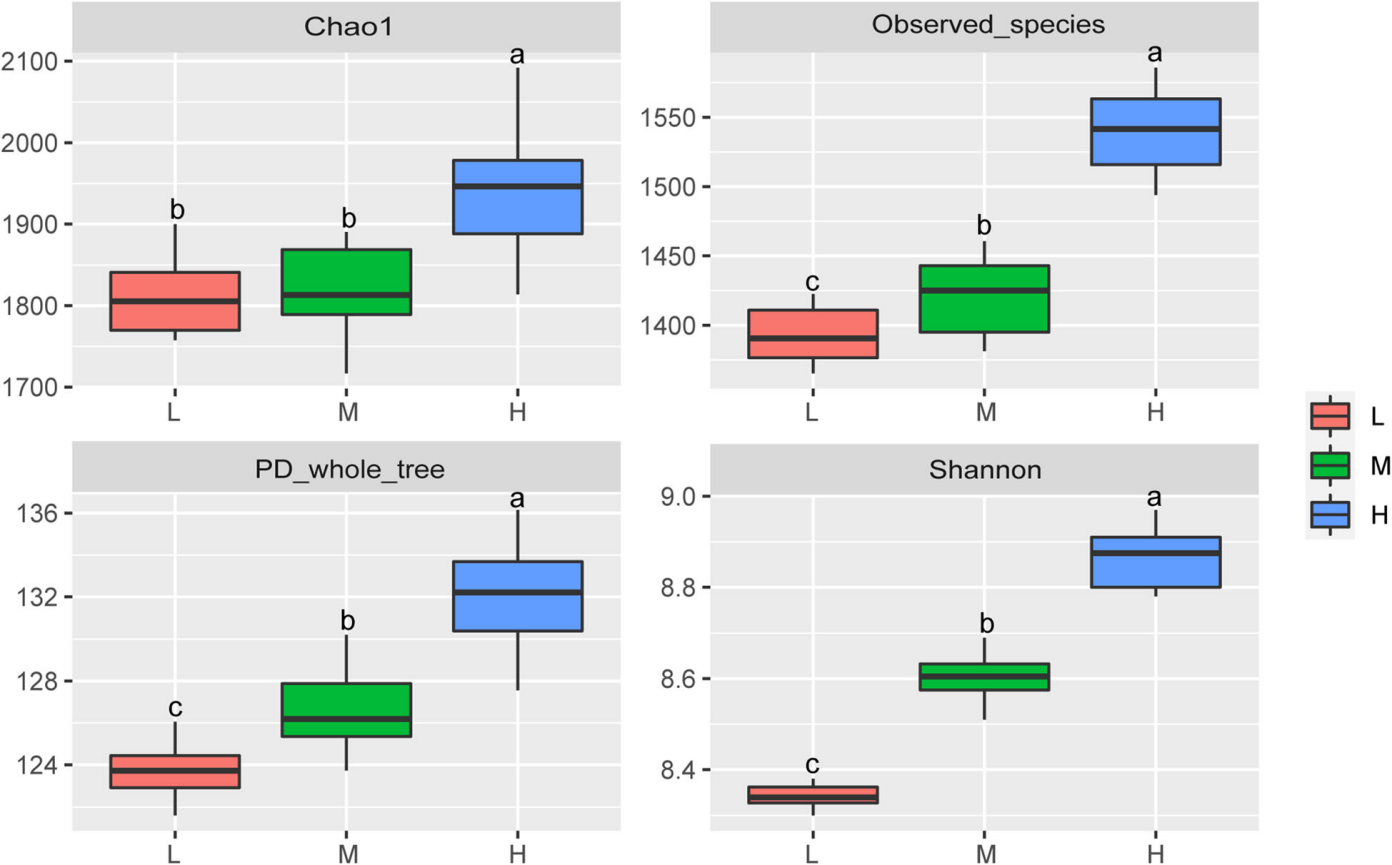
Community richness estimates and diversity indices for the three elevations. ^a, b, c^ Boxes with different superscripts differ significantly (*P* < 0.05). L, 2800 m; M, 3700 m; H, 4700 m

A total of 2,833,238 raw reads were obtained from 36 samples, with an average of 78,701 reads for each sample (minimum, 45,511; maximum, 113,622). Using these sequences, we identified 2894 OTUs based on 97% nucleotide sequence identification between total reads. A total of 1701 OTUs were shared between samples from different elevations; the total OTUs of the high, middle, and low altitude samples were 2446, 2302, and 2279, respectively (Fig. 1a). Taxonomic analysis of the reads revealed 22 bacterial phyla. *Firmicutes* and *Bacteroidetes* were the predominant phyla, accounting for 52.31 and 37.08% of the total sequences, respectively (Fig. 2a). *Actinobacteria*, *Verrucomicrobia*, and *Proteobacteria* represented 3.27, 1.36, and 1.01% of the total sequences, respectively. The alpha diversity index analysis is shown in Fig. 3. The community diversity indices (Shannon index) enhanced with altitude (*P* < 0.05). The community richness counts (Chao 1 estimator) at high altitude were greater than at middle and low altitudes (*P* < 0.05). PCoA plots based on weighted UniFrac distance metric revealed the differences in microbial diversity between samples from the three elevations (Fig. 1b).

The effects of altitude on the prevalence of certain bacterial phyla (average relative abundance ≥1% for at least one elevation) in yak rumen are presented in Table S2. The relative abundance of *Firmicutes* increased (*P* < 0.05), whereas the proportion of *Bacteroidetes* decreased (*P* < 0.05) with altitude. Yaks at the middle altitude had a higher relative abundance of *Actinobacteria* (*P* < 0.05) compared with yaks at high and low altitudes. At the family level, *Ruminococcaceae* (18.86%), *Prevotellaceae* (15.04%), *Christensenellaceae* (13.69%), and *Lachnospiraceae* (10.04%) were the dominant families; other families included *Bacteroidales_BS11_gut_group* (8.74%), *Rikenellaceae* (7.72%), and *Coriobacteriaceae* (3.19%) (Fig. 2b). The relative abundances of *Ruminococcaceae* and *Christensenellaceae* increased with altitude, while the relative abundance of *Prevotellaceae* decreased with altitude (Table S3). The relative abundances of the *Coriobacteriaceae* and *Rikenellaceae* families in M were higher than the abundancies in H and L (*P* < 0.05). At the genus profile level, 208 taxa were identified, and the proportions of 25 genera (average relative abundance ≥0.1% for at least one elevation) differed between the three altitudes (Table S4). Among these, *Prevotella_1* (11.57%) was the most dominant genus, followed by *Christensenellaceae_R-7_group* (9.87%), *Rikenellaceae_RC9_gut_group* (6.83%), and *Ruminococcaceae_NK4A214_group* (5.83%) (Fig. 2c). The relative abundances of *Ruminococcus_1*, *Christensenellaceae_R-7_group*, *Romboutsia*, *Alloprevotella*, *E. coprostanoligenes*, *Clostridium*, *Treponema*, and *Streptococcus* increased with altitude (*P* < 0.05), however, the proportions of *Prevotellaceae_UCG-001*, *Succiniclasticum*, *Butyrivibrio_2*, and *Lachnospiraceae_XPB1014_group* decreased (*P* < 0.05). The relative abundances of the genera, *Rikenellaceae_RC9_gut_group*, *Oribacterium*, *Saccharofermentans*, *and Ruminococcaceae_UCG-014*, at the middle altitude were higher than at the low and high altitudes (*P* < 0.05).

**Fig. 4:**
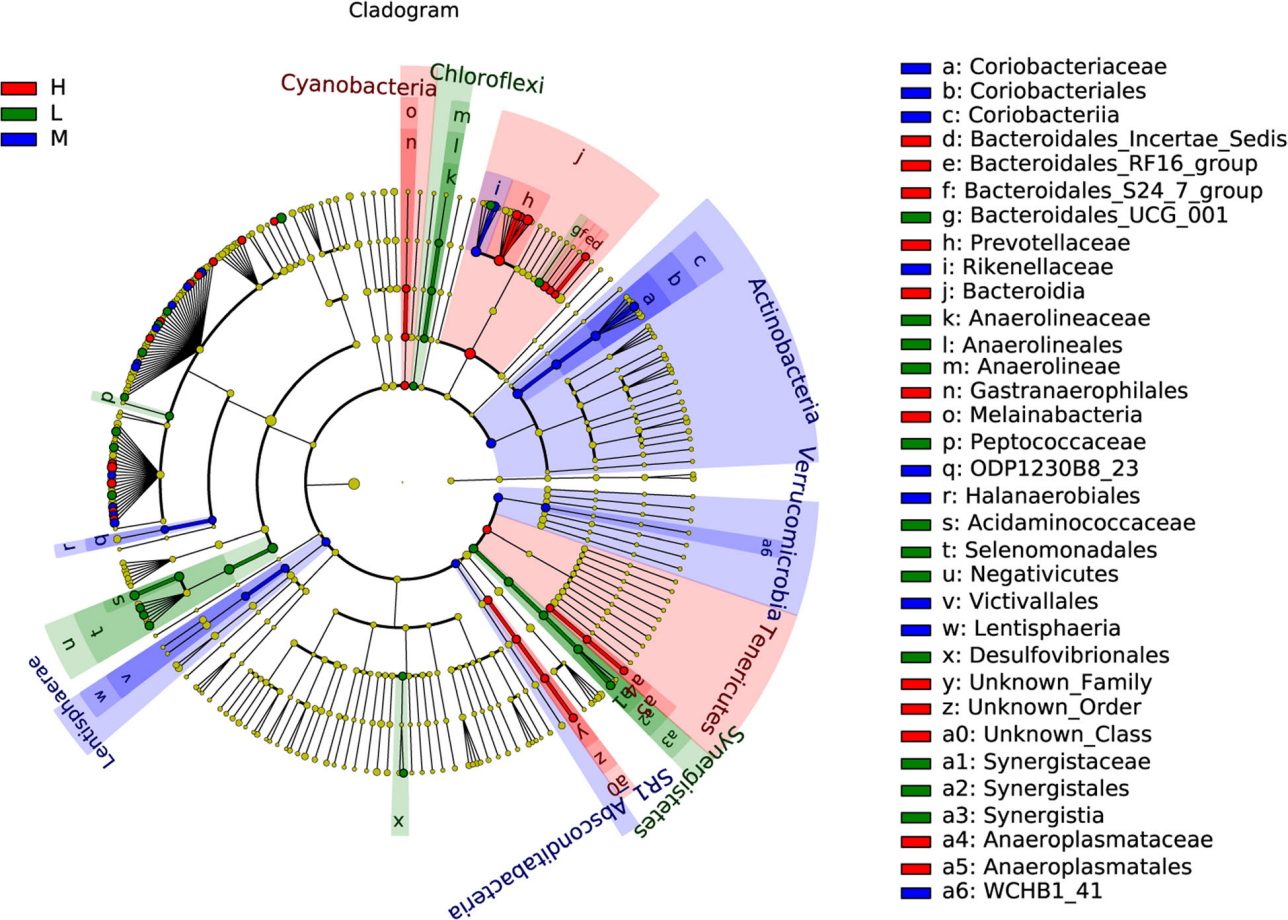
LEfSe (Linear discriminant analysis Effect Size) cladogram comparing microbial communities among the three elevations. Differences are represented by the color of the group where taxa are most abundant; Red: taxa abundant in H, Green: taxa abundant in L, Blue: taxa abundant in M. L, 2800 m; M, 3700 m; H, 4700 m

We also performed LEfSe (Linear discriminant analysis Effect Size) to detect variations in the bacterial taxa composition. Figure 4 depicts a representative cladogram of the structure of the predominant microbiome, showing the most remarkable differences in taxa among the different elevations. The data indicated that twelve clades were more abundant in the L group, nine clades were more abundant in the M group, and twelve clades were more abundant in the H group. The different bacterial taxa from the three elevations are shown in Fig. S1. Moreover, when the microbial communities were compared in the context of different elevations, the most differentially abundant bacterial genera in L were *Christensenellaceae_R-7_group* and *Ruminococcaceae_UCG-010*, *Butyrivibrio_2*; *Ruminococcus_1* was more abundant in M, while *Prevotella_1* and *Ruminococcaceae_NK4A214_group* were more abundant in H. Of note, the genera *Butyrivibrio_2* and *Prevotella_1* were the most differentiated among communities, with an absolute LDA score factor of ~ 5.

### Correlations between bacterial communities and rumen fermentation parameters

**Fig. 5:**
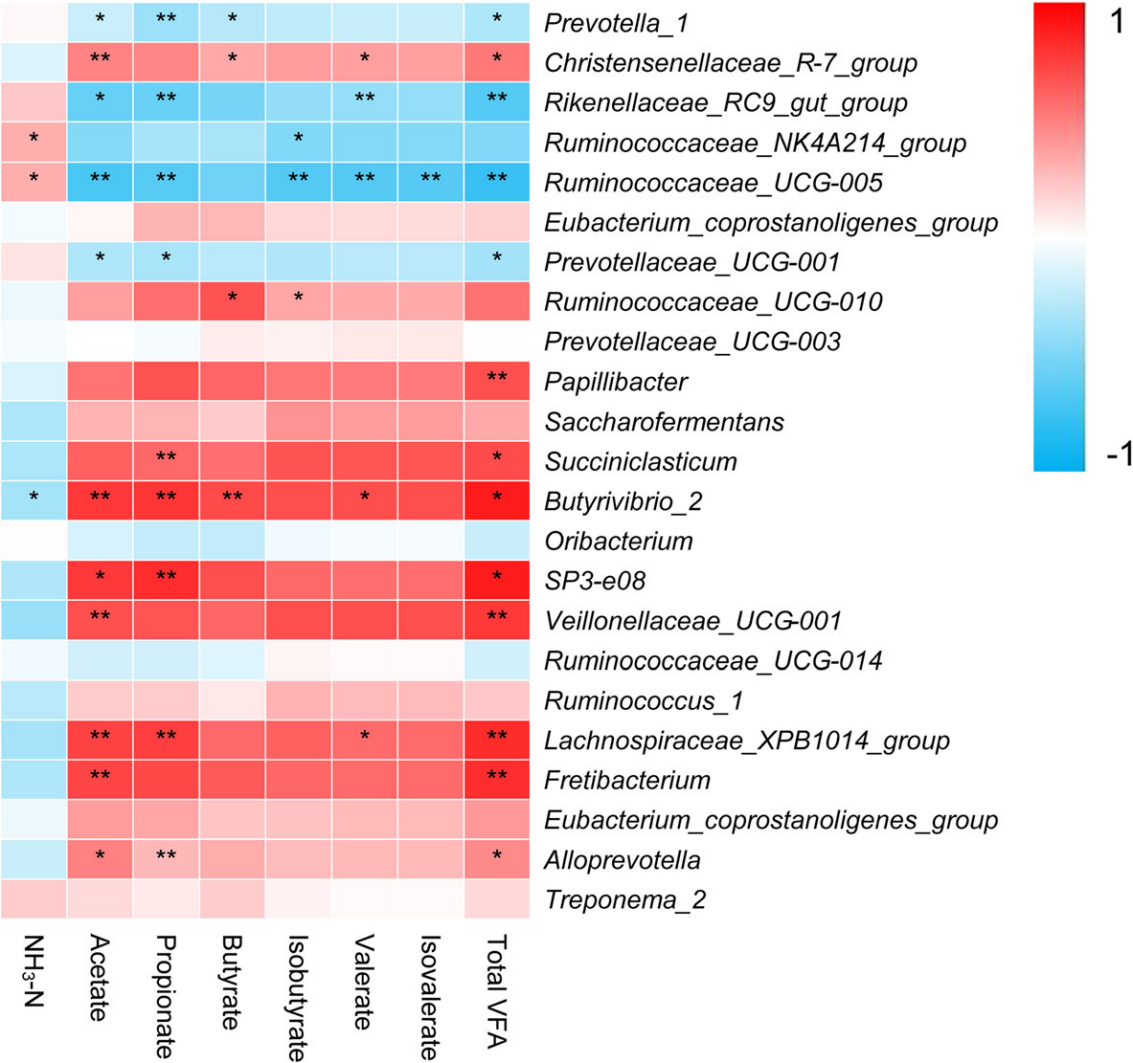
Correlation between the relative abundances of rumen bacteria and fermentation parameters. “*” and “**” indicate the significance level at 0.05 and 0.01, respectively

We analyzed the correlation between the relative abundance of rumen bacteria genera and fermentation parameters through correlation analysis (Fig. 5). The abundances of the rumen bacterial genera and the rumen NH_3_-N and TVFA concentrations were closely related to each other if *P* < 0.05. The NH_3_-N concentration was positively correlated with the relative abundances of genera *Ruminococcaceae_NK4A214_group* and *Ruminococcaceae_UCG-005*, and were reversely correlated with *Butyrivibrio_2* abundance. The acetate molar proportion was positively correlated with the relative abundance of genera *Veillonellaceae_UCG-001*, while *Alloprevotella* was negatively correlated with *Prevotella_1*, *Rikenellaceae_RC9_gut_group*, and *Prevotellaceae_UCG-001* abundances. The propionate molar proportion was positively correlated with *Alloprevotella* and *Succiniclasticum* and was reversely correlated with *Prevotella_1* and *Prevotellaceae_UCG-001* abundances. The butyrate molar proportion was positively correlated with *Butyrivibrio_2*, *Ruminococcaceae_UCG-010*, together with *Christensenellaceae_R-7_group*, and was negatively associated with *Prevotella_1* abundance. The isobutyrate molar proportion was positively correlated with *Ruminococcaceae_UCG-010* and was reversely associated with *Ruminococcaceae_NK4A214_group* abundance. The valerate molar proportion was positively associated with *Christensenellaceae_R-7_group*, *Butyrivibrio_2*, and *Lachnospiraceae_XPB1014_group*, and was negatively correlated with *Rikenellaceae_RC9_gut_group* abundance. The TVFA concentration was directly associated with the relative abundances of the genera *Christensenellaceae_R-7_group*, *Succiniclasticum*, *Butyrivibrio_2*, and *Alloprevotella*, and was negatively correlated with *Prevotella_1* and *Prevotellaceae_UCG-001* abundances.

### Tax4Fun gene function estimation

**Fig. 6:**
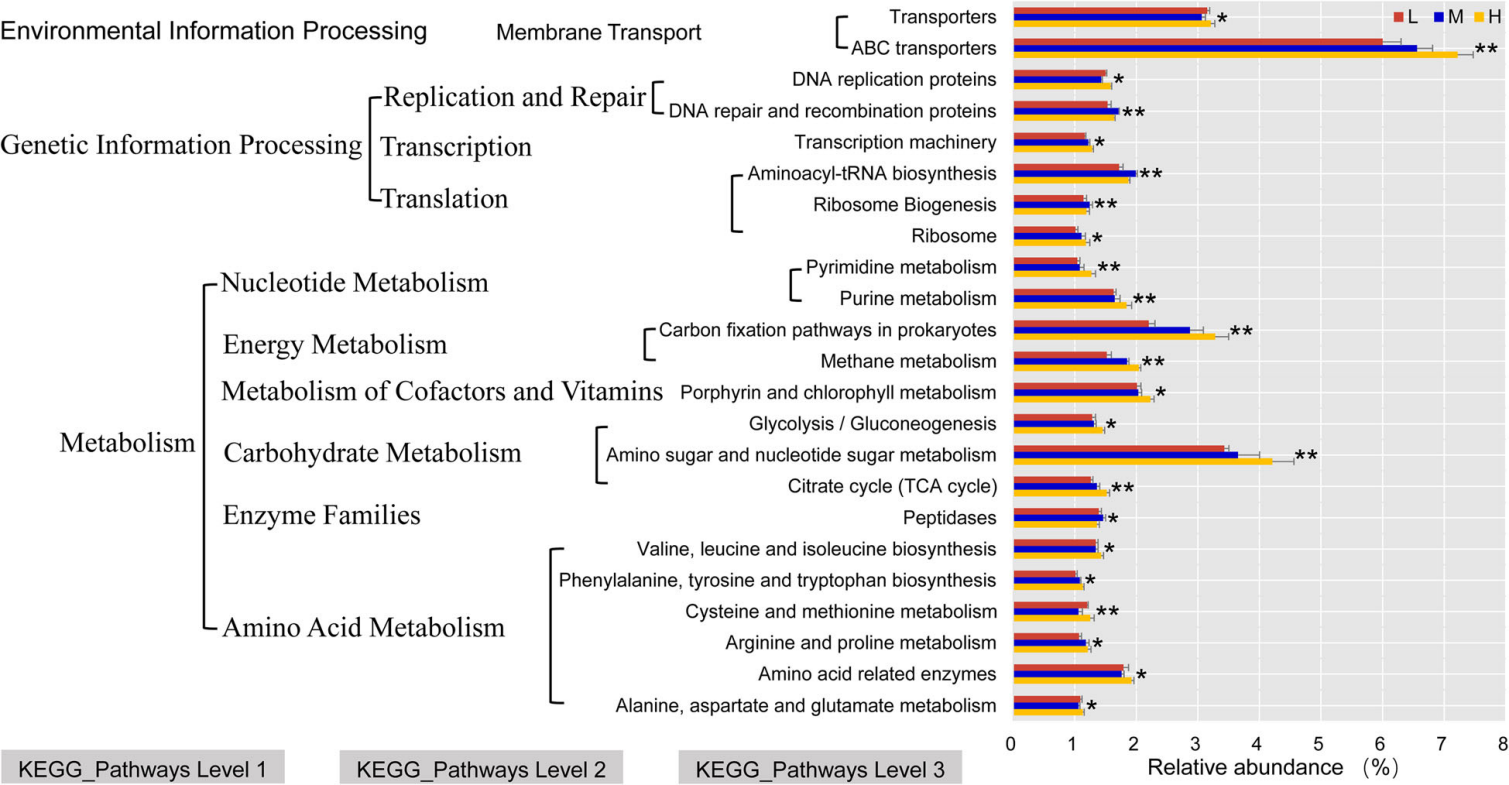
Functional predictions for rumen microbiota with significantly different KEGG pathways (*P* < 0.05) for the three elevations (H, M, and L). KEGG pathways at Level 1, Level 2, and Level 3 are represented. L, 2800 m; M, 3700 m; H, 4700 m. “*” and “**” indicate the significance level at 0.05 and 0.01, respectively

Tax4Fun was used to predict the function of the rumen microbiota of yaks from three altitudes. Interestingly, the relative abundance of ABC transporters (6.63%) was the highest at all the three altitudes, and amino sugar and nucleotide sugar metabolism (3.77%) was the second-most abundant. The Tax4Fun predictive software enriched 46 predominant pathways (relative abundances > 1%) in the level 3 KEGG pathways. Among these, 23 pathways showed significant differences at high, middle, and low altitudes (*P* < 0.05) (Fig. 6). Notably, the relative abundances of carbohydrate and energy metabolism gene categories significantly increased with altitude (*P* < 0.05).

## Discussion

Biodiversity is imperative in promoting the sustainability and productivity of numerous ecosystems [15]. Therefore, high microbial diversity is closely related to strong metabolic ability and stability [11]. The diversity of human intestinal microbiota improves the fermentation efficiency of dietary fiber and promotes stability of the intestinal ecosystem [16]. In addition, the increase in diversity of human gut microbes also reflects better health and stronger metabolic capacity [17]. In this study, rumen microbial diversity of yaks increased with altitude, and was comparable to the gastrointestinal tract (GIT) microbial diversity of other mammals, such as pikas [11] and rhesus macaques [18]. Therefore, we speculate that the rumen communities of high-altitude yaks may have a higher ability to use high-fiber herbage to help them meet their energy needs in cold and high-altitude habitats.

This study revealed that the phyla *Firmicutes* and *Bacteroidetes* were the predominant bacteria in the rumen of yaks. The two phyla in the current study were also found to be abundant in the GIT of yaks [1, 2, 4], sheep [19], goats [20], bovine animals [21], and pikas [11], indicating an ecological and functional role of *Firmicutes* and *Bacteroidetes* in the mammalian GIT. Huang and Li (2017) [22] reported that the relative abundance of *Firmicutes* in yaks was higher than that of *Bacteroidetes* during grazing. The same conclusion was made in this study. *Firmicutes* perform essential functions in energy conversion and harvesting [23], whereas *Bacteroidetes* are responsible for protein hydrolysis, carbohydrate degradation, and fermentation of amino acids to acetate [24]. In the current study, the relative abundance of *Firmicutes* increased with altitude, whereas the proportion of *Bacteroidetes* decreased. The increase in *Firmicutes* and the ratio of *Firmicutes* to *Bacteroidetes* in rumen indicates that yaks grazing in high elevation pastures may possess better herbage energy utilization ability and increased resistance to cold stress. Similar findings were also found in mice exposed to cold environments. The non-shivering thermogenesis and energy harvest of these mice increased, signaling that high altitude yaks may have a higher energy harvest and consumption [25].

At the family level, the relative abundances of *Ruminococcaceae* and *Christensenellaceae* increased with altitude, while the relative abundance of *Prevotellaceae* decreased. In an earlier study, *Ruminococcaceae* played essential roles in cellulose degradation in the GIT [26]. Through rumen fermentation, cellulose can be degraded by microorganisms into VFA, which is an essential energy source for epithelial cells and can provide 60–75% of the required metabolic energy to the host [27]. In black howler monkeys, the abundance of *Ruminococcaceae* increased during periods of energy deprivation and seemed to compensate for the decrease in energy intake [28]. In high-altitude habitats, yaks suffering from low oxygen and cold environments need more energy to maintain their metabolic balance, but the available food sources are limited [10]. Therefore, an increased abundance of *Ruminococcaceae* may lead to increased energy utilization efficiency to support yaks living in cold and high-altitude environments. *Christensenellaceae* can naturally secrete α-arabinosidase, β-galactosidase, and β-glucosidase, which are also closely related to feed efficiency [29]; hence, it is speculated that these bacteria are vital for the adaptation of high-altitude yaks to the harsh environment on the QTP. Stewart et al. (2018) [30] identified rumen uncultured genomes with large amounts of polysaccharide utilization loci associated with the *Prevotellaceae* family that contain proteins capable of binding and degrading a variety of carbohydrate substrates. Li and Zhao (2015) [31] revealed that the relative abundance of *Prevotellaceae* was decreased in the gut of Han Chinese living in Tibet. These results suggest a relevant role of *Prevotellaceae* in the high-altitude adaptation. However, a previous study showed that the relative abundance of *Prevotellaceae* was increased in the gut of Plateau pika (4431 m elevation) [32]. These differences imply that various species may have an adaptable gut microbiota composition as an adaptation to high-altitude environments. In addition, dietary composition is another key factor affecting the GIT microbiota [13]; therefore, comparing the rumen microbiota of yaks fed the same diet at different altitudes may further enhance the understanding of the role of microbiota in host adaptation.

Importantly, the relative abundances of *Ruminococcus_1*, *Christensenellaceae_R-7_group*, *Romboutsia*, *Alloprevotella*, *E. coprostanoligenes*, *Clostridium*, *Treponema*, and *Streptococcus* clearly increased with altitude, demonstrating that these microorganisms in the rumen may adapt well to low temperature and low oxygen environments. The microorganisms that were enhanced in the high-altitude yaks may be involved in performing important functions for the host. For example, *Christensenellaceae_R-7_group* and *Ruminococcus_1* contain genes for essential cellulase and hemicellulase secreting enzymes [33, 34] that may improve the yak’s ability to degrade plant cellulose and obtain energy from indigestible polysaccharides. The genus *Romboutsia* has a variety of metabolic abilities, which can be engaged in the fermentation of carbohydrates and the utilization of single amino acids [35]. *Alloprevotella* is reported to be closely related to decreased cardiovascular disease risk [36], and can produce moderate concentrations of rumen acetate, but major amounts of succinate [37]. This result is inconsistent with the present study showing a positive correlation between the relative abundance of *Alloprevotella* and acetate and propionate concentrations. *E. coprostanoligenes* is documented to have a cholesterol- removing capability [38]. Additionally, a number of organisms in the genus *Clostridium* are associated with cellulose degradation and nitrogen fixation [39]. This study demonstrated that some potential and relevant probiotics were significantly enhanced in the high-altitude grazing yaks. These genera in the rumen may help yaks improve metabolic capability and maintain host health to consequently tolerate to the harsh environments at higher elevations. Nevertheless, more investigations are warranted to clearly evaluate the ecological functions of these bacteria in yak rumen.

In addition to the observed potential probiotics, we found that a potentially pathogenic bacteria, *Treponema*, also increased with elevation. Some species of *Treponema* have been detected in the genital and oral tracts of humans [40], the rumen of yaks [41], and the gut of rhesus macaques [18]. They can cause various diseases in these organisms, such as syphilis, yaws, and papillomatous digital dermatitis [18, 42]. Despite the potential risk of these pathogens, their functional roles in yaks still remain unclear and deserve further investigation.

Along with the potential roles of probiotics and pathogens, it was found that commensal bacteria were enriched in the rumen of high-altitude yaks. *Streptococcus* is a common group of bacteria found in human skin, mouths, throats, and intestines [43]. The genus *Streptococcus* has also been detected in pika mouths [32] and gut [11], indicating that this genus may be widely distributed in mammalian host microbiota. Additionally, some species of this genus have been found to be related to human pyruvate metabolism [44]. Therefore, we speculate that they play a key role in the rumen of high-altitude yaks.

In the current study, some bacteria, including *Christensenellaceae_R-7_group*, *Ruminococcaceae_UCG.010*, *Succiniclasticum*, *Butyrivibrio_2*, and *Alloprevotella*, were found closely associated with VFAs. It is difficult to conclude which genus was responsible for the specific VFA due to the complex interactions among microbes, such as resource competition [45] and cross-feeding [46], but these bacteria likely play key roles in the fermentation of herbage structural carbohydrates in the yak rumen.

In addition to variations of yak rumen microbiomes, VFAs exhibited remarkable fluctuations in response to altitude. VFAs are produced by rumen microorganisms fermenting plant cellulose and other carbohydrates, and play an essential role in ruminant growth and immunity [47]. VFAs formed in the rumen are largely absorbed across the host’s ruminal epithelium [9, 48]. Thus, the high altitude yaks might possess the ability to more efficiently transport and absorb VFAs than those grazing at low altitude. Acetic and propionate acid could activate the GPR43 and GPR41 receptors to produce PYY and GLP-1 hormones, which can increase glucose utilization for body energy [49]. It is worth noting that the main VFA (acetic acid) in the rumen may reduce the abundance of *Escherichia coli*, thereby maintaining rumen health [50]. This research showed that TVFA concentration and the proportions of acetate and propionate significantly increased with altitude, indicating that high altitude yaks were more capable to thrive under the harsh conditions of Tibetan pastures compared to those at the middle and low altitudes. Thorough assessment of plasma VFA levels are needed to reinforce the finding related to VFA transport in high-altitude ruminants. Such analyses may provide valuable insights for understanding how changes in the microbiota and host genes are related to each other.

Microorganisms have an impact on the body’s immunity, degradation and absorption of nutrients, and even enzyme metabolism [5]. In the current study, we utilized Tax4Fun to predict the function of the yak rumen microbial community. Our data indicates that the estimated gene functional profiles of yak rumen microbiomes were significantly impacted by altitude. Most dramatically, in KEGG pathways level 2, those genes involved in carbohydrate and energy metabolism were enhanced in the high altitude yaks. This indicates that the rumen microbiota of the high-altitude yaks produce a large amount of VFAs to provide the host with additional energy, helping the host to maximize the use of nutrients and indigestible plant components, such as cellulose. Although high altitude yaks ingest higher fiber herbage as compared to middle and low altitude yaks, they may more efficiently degrade high-fiber herbage due to improved rumen microbial diversity and function. Thus, the enhanced ability of high-altitude yaks to utilize herbages may be a kind of microbiota adaptation for more energy requirements in cold and hypoxic high-altitude environments. Among the KOs, high-altitude yaks showed enrichment in the carbon fixation pathways of prokaryotes; this was consistent with the highly efficient formation of VFAs [51]. Notably, ABC transporters were the most expressed pathway in membrane transport and directly participate in the production of ATP. Furthermore, Hamana et al. (2012) [52] revealed that ABC transport function is a barrier to protect ruminants from the invasion of toxic substances. In this study, ABC transporters were expressed at a significantly higher level in high altitude yaks. In a high-altitude environment, low oxygen and high ultraviolet radiation may cause DNA and protein damage, and genes related to replication and repair may help reduce damage to biomolecules. Therefore, this pathway may help yaks adapt to high-altitude environments. However, our results were only based on the predicted metagenomics, and may not represent the actual function of rumen bacteria. Further studies should be conducted to directly sequence the yak rumen metagenome to explore the roles of these genes in yak environmental adaptability. Future studies using metagenome analysis are also needed to explore the roles of these gene functions in yak environmental adaptability.

## Conclusions

Our results showed that some potential probiotics, including *Christensenellaceae_R-7_group*, *Ruminococcus_1*, *Romboutsia*, *Alloprevotella*, *E. coprostanoligenes*, and *Clostridium*, were enriched in the rumen of high-altitude yaks. Shifts in the rumen microbiomes were caused by a high-altitude environment characterized by cold temperatures, hypoxia, and the production of high-fiber herbage. Moreover, rumen microbial diversity and herbage fermenting ability of yaks increased with elevation; therefore, in high altitude yaks, these should be considered as microbiota adaptation to partially meet the higher energy requirements needed for survival in the harsh cold and hypoxic environment.

## Methods

### Animals and sample collection

**Fig. 7:**
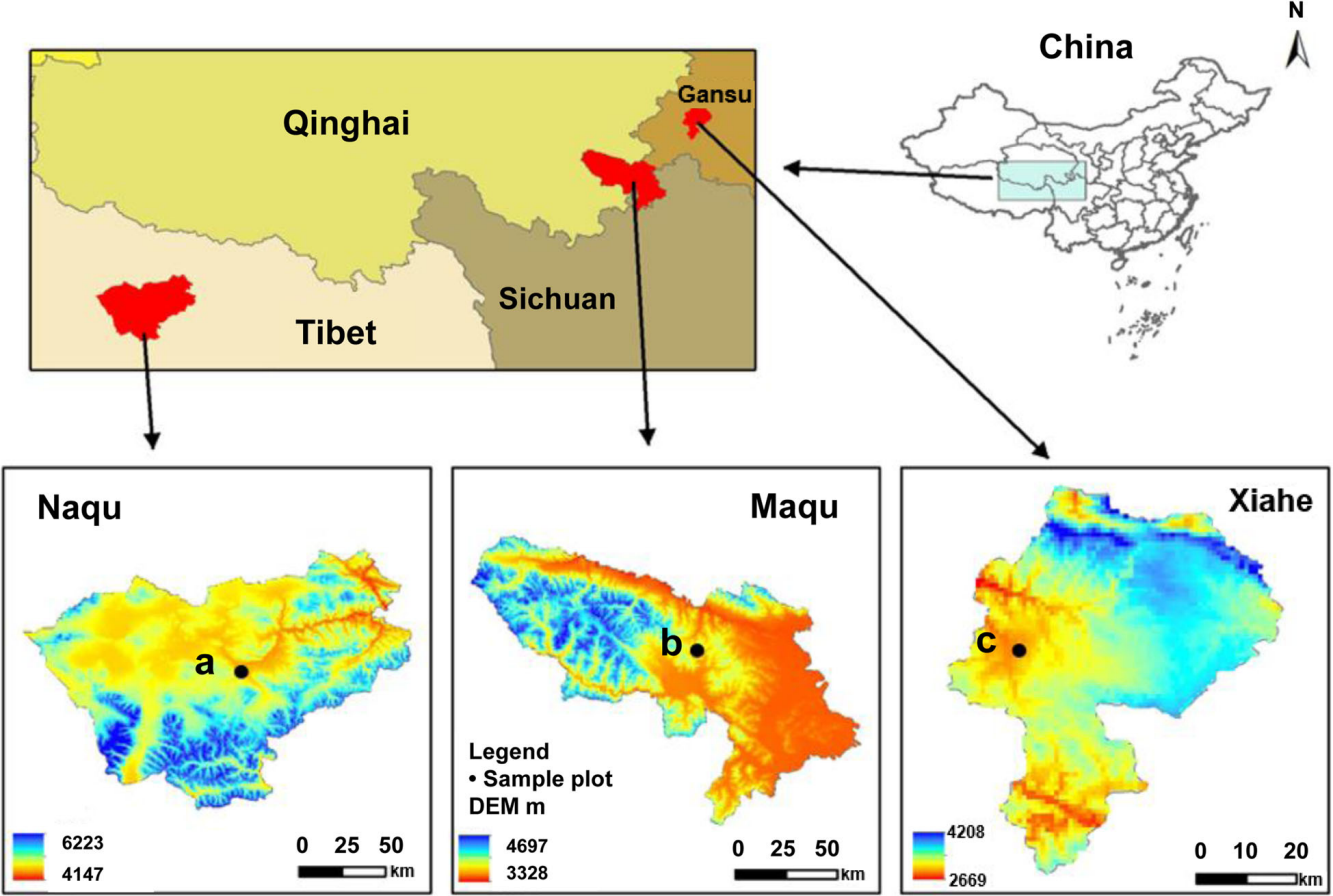
Sampling sites of herbage and yaks from 3 different altitudes in the Qinghai-Tibet plateau. a, Sangke Township (Xiahe County, Gansu Province, 2800 m); b, Manrima village (Maqu County, Gansu Province, 3700 m); c, Gulu Township (Naqu Prefecture, Tibet Autonomous Region, 4700 m)

Three districts in the middle part of the northwestern region of QTP of China were selected as experimental sites (Fig. 7): Sangke Township (latitude 34°17′36″N, longitude 102°18′31″E; altitude 2800 m), Xiahe County, Gansu Province; Manrima village (latitude 33°40′4″N, longitude 101°52′12″E; altitude 3700 m), Maqu County, Gansu Province; and Gulu Township (latitude 30°58′68″N, longitude 91°37′34″E; altitude 4700 m), Nagqu Prefecture, Tibetan Autonomous Region. These three locations are typical areas for raising the yaks on the QTP, with mean annual air temperatures of 1.2 °C, − 0.5 °C, and − 1.5 °C, respectively, and a mean annual precipitation of 620 mm, 518 mm, and 422 mm, respectively. The vegetation consisted of typical alpine meadows. The main edible herbage species and the proportions are presented in Table S5. At the study sites, yaks commonly grazed in a full-grazing system with herbage as the only feed [10]. We selected 12 healthy yaks 5 years of age with an average initial body weight of 284.38 ± 8.36 kg from each sampling site. Rumen fluid samples and herbage samples were collected during the summer in mid July at Sangke Township, late July at Manrima village, and early August at Gulu Township. Ten-day intervals were set so that the herbage would be approximately in the same phenological stage.

### Sampling of plants and analyses of chemical composition

A total of 20 randomly selected quadrats (50 cm × 50 cm) were chosen from the vegetation on which the yaks grazed. Mixed herbage samples were collected in the quadrats, inedible herbage was removed, and only edible herbage was retained. The samples were placed in a 60 °C oven for 24 h to a constant weight, ground using a mill, and passed through a 1 mm sieve for further chemical analysis. The dry matter (DM) of the herbage was prepared by subjecting the samples for dry matter determination in an air-flow oven at 65 °C for 72 h [53]. Nitrogen content was determined by the Kjeldahl method and crude protein (CP) concentration was calculated as 6.25 × N. Ether extract (EE) was measured by the weight loss of dry matter after 8 h of extraction with ether in a Soxhlet extractor [53]. The fibrous fractions of neutral detergent fiber (NDF) and acid detergent fiber (ADF) contents were analyzed using the methods outlined by Van Soest et al. (1991) [54].

### Sampling of rumen digesta and analyses of fermentation parameters

Twelve yaks of similar average body weight in each sampling site were selected for collection of ruminal content (liquid and particulate forage material), which was sampled in the morning before grazing. All animals in this experiment continued to graze on natural grass after collecting rumen fluid and were in good health condition. Samples (approximately 50 mL) were collected using an oral stomach tube as described by Fan et al. (2020) [55]. The first 50 mL of rumen fluid was discarded to avoid contamination from previous animas or its own saliva [4]; this was followed by collection of 50 mL rumen fluid from each animal, and immediate pH measurement by pH meter (Model 144 PB-10, Sartorius Co., Germany). The rumen contents were filtered with four layers of woven gauze and divided into two portions for analysis of ruminal fermentation parameters and for DNA extraction. For analysis of VFA concentrations, the filtrate was thawed and centrifuged at 1000×g for 15 min and then analyzed using gas chromatography (chromatograph SP-3420A, Beifenrili Analyzer Associates, Beijing, China) as described by Erwin et al. (1961) [56]. The concentration of NH_3_-N in the rumen was later analyzed using a specific visible spectrophotometry device (UV-VIS8500, Tianmei, Shanghai, China) [57].

### DNA extraction, sequencing, sequence processing, and analysis

Bacterial DNA was prepared and extracted from the digesta using an E.Z.N.A.® Stool DNA kit (Omega Bio-TEK, Norcross, GA, USA). The concentration and purity of the extracted DNA were detected using an ultra-microspectrophotometer (NanoDrop 2000C, Thermo Scientific, USA). The V3-V4 region of the 16S rRNA was amplified using primers 338F (5-ACTCCTACGGGAGGCAGCAG-3) and 806R (5- GGACTACHVGGGTWTCTAAT-3) [58]. The barcode of the unique eight-base sequence of each sample was added to each primer for sample identification and determination. PCR was conducted in triplicates as follows: an initial denaturing step at 94 °C for 5 min, followed by 28 cycles at 94 °C for 30 s, 55 °C for 30 s, and 72 °C for 60 s, and a final extension at 72 °C for 7 min. Amplicons were extracted from 2% agarose gels, purified using the AxyPrep DNA Gel Extraction Kit (Axygen Biosciences, Union City, CA, USA) according to the manufacturer’s instructions, and quantified using the QuantiFluor™-ST system (Promega, Madison, WI, USA). Purified amplicons were pooled in equimolar concentrations and paired-end sequenced (2 × 300 bp) on an Illumina MiSeq PE300 platform (Illumina, Inc., San Diego, CA, USA) according to the standard protocols. The processing of the sequencing data was mainly performed using QIIME 1.9.0 software [59]. The original sequences were sorted based on their unique sample barcodes and were trimmed for sequence quality using the QIIME pipeline (length > 300 bp, average base quality score > 30) [60]. Chimera sequences were removed with the UCHIME algorithm. These effective tags were clustered into operational taxonomic units (OTUs) based on a sequence similarity threshold of 97% using UPARSE (version 7.0) [61]. Representative sequences were classified into organisms using RDP classifier (version 2.2) based on the SILVA (SSU123) database [62]. Alpha diversity analysis was performed by calculating the Chao1 index, Shannon index, phylogenetic diversity index (PD_whole_tree), and observed species index (observed_species) using QIIME (version 1.9.0). The PCoA with weighted UniFrac distance matrices and the analysis of similarity in QIIME were used to estimate differences in bacterial communities between samples [63].

### Statistical analysis

The chemical composition of herbage, ruminal fermentation parameters, relative abundance of bacteria, and the alpha diversity indices were analyzed using a completely randomized design by one-way analysis of variance (SAS Institute Inc., version 9.2, USA). Significant difference was declared at *P* < 0.05. Pearson correlation coefficients between bacterial consortium and rumen fermentation end-products parameters were calculated using the PROCCORR procedure of SAS 9.2 with a heatmap format as described by Pan et al. (2017) [64]. Briefly, only those bacterial taxa with an abundance > 0.1% of the total consortium in at least one ruminal sample were used in the analysis. The abundances of bacterial consortium at the genus level and ruminal parameters were considered to be correlated with each other for correlation coefficient values (|r|) ≥ 0.55 and *P <* 0.05 [65]. Tax4fun software was used to compare the species compositions obtained from the 16S sequencing data and then to infer the functional gene composition of samples. The functional composition was predicted from the Kyoto Encyclopedia of Genes and Genomes (KEGG) database [66].

## Supplementary Information

**Additional file 1: Table S1.** Nutrient composition of herbage at different altitudes

**Additional file 2: Table S2.** Comparison of the dominant phyla (average relative abundance ≥1% for at least one altitude) within the rumen

**Additional file 3: Table S3.** Comparison of the dominant families (average relative abundance ≥1% for at least one altitude) within the rumen

**Additional file 4: Table S4.** Comparison of the dominant genera (average relative abundance ≥0.1% for at least one altitude) within the rumen

**Additional file 5: Table S5.** Major species and proportion of edible herbage at different altitudes

**Additional file 6: Figure S1.** Histogram of LDA scores computed for each taxon ranging from phylum to genus. L, 2800 m; M, 3700 m; H, 4700 m; LDA, linear discriminant analysis

## Data Availability

The datasets used and/or analysed during the current study are available from the corresponding author on reasonable request.

## Abbreviations

OTUs: Operational taxonomic units
PCoA: Principal coordinate analysis
QIIME: Quantitative insights into microbial ecology
VFA: Volatile fatty acids
NH_3_-N: Ammonia nitrogen
PCR: Polymerase chain reaction
DNA: Deoxyribonucleic acid
rRNA: Ribosomal ribonucleic acid
KEGG: Kyoto Encyclopedia of Genes and Genomes
LEfSe: Linear discriminant analysis Effect Size
LDA: Linear discriminant analysis
