# Supplementary material for: Altitude influences microbial diversity and herbage fermentation in the rumen of yaks
Source: BMC Microbiol. 2020 Dec 4;20:370. doi: 10.1186/s12866-020-02054-5 (PMC7718673; doi:10.1186/s12866-020-02054-5)
Supplement: Supplementary file 1 — Additional file 1: Table S1. Nutrient composition of herbage at different altitudes [file 12866_2020_2054_MOESM1_ESM.docx]

| Chemical composition ^1^ | Altitude ^2^ | | | SEM ^3^ | *P* value |
| --- | --- | --- | --- | --- | --- |
|  | L | M | H |  |  |
| DM | 93.57 | 93.61 | 93.56 | 0.0319 | 0.7589 |
| OM | 89.61 | 89.71 | 89.68 | 0.0662 | 0.8333 |
| CP | 11.22 | 10.64 | 9.93 | 0.2582 | 0.1208 |
| EE | 3.37 | 3.30 | 3.32 | 0.0266 | 0.6201 |
| NDF | 53.16^c^ | 58.93^b^ | 65.63^a^ | 1.0619 | 0.0325 |
| ADF | 31.29^b^ | 28.79^c^ | 34.35^a^ | 0.3898 | 0.0132 |

^a,b,c^ Values in the same row with different superscript letters differ significantly (*P* < 0.05)

^1^ DM, dry matter; OM, organic matter; CP, crude protein; EE, ether extract; NDF, neutral detergent fiber; ADF, acid detergent fiber

^2^ L, 2,800 m; M, 3,700 m; H, 4,700 m

^3^ Standard error of the mean
