# Supplementary material for: Altitude influences microbial diversity and herbage fermentation in the rumen of yaks
Source: BMC Microbiol. 2020 Dec 4;20:370. doi: 10.1186/s12866-020-02054-5 (PMC7718673; doi:10.1186/s12866-020-02054-5)
Supplement: Supplementary file 2 — Additional file 2: Table S2. Comparison of the dominant phyla (average relative abundance ≥1% for at least one altitude) within the rumen [file 12866_2020_2054_MOESM2_ESM.docx]

| Phylum | Altitude ^1^ | | | SEM ^2^ | *P* value |
| --- | --- | --- | --- | --- | --- |
|  | L | M | H |  |  |
| *Firmicutes* | 48.87^c^ | 52.42^b^ | 55.63^a^ | 0.7513 | 0.0003 |
| *Bacteroidetes* | 41.64^a^ | 35.86^b^ | 33.73^b^ | 0.7835 | < 0.0001 |
| *Actinobacteria* | 2.91^b^ | 4.67^a^ | 2.22^b^ | 0.2492 | < 0.0001 |
| *Verrucomicrobia* | 1.11 | 1.69 | 1.28 | 0.1574 | 0.1245 |
| *Proteobacteria* | 0.89 | 0.97 | 1.17 | 0.2327 | 0.2823 |
| *Firmicutes: Bacteroidetes* | 1.19^c^ | 1.48^b^ | 1.68^a^ | 0.0493 | < 0.0001 |

^a,b,c^ Values in the same row with different superscript letters differ significantly (*P* < 0.05)

^1^ L, 2,800 m; M, 3,700 m; H, 4,700 m

^2^ Standard error of the mean
