# Supplementary material for: Altitude influences microbial diversity and herbage fermentation in the rumen of yaks
Source: BMC Microbiol. 2020 Dec 4;20:370. doi: 10.1186/s12866-020-02054-5 (PMC7718673; doi:10.1186/s12866-020-02054-5)
Supplement: Supplementary file 3 — Additional file 3: Table S3. Comparison of the dominant families (average relative abundance ≥1% for at least one altitude) within the rumen [file 12866_2020_2054_MOESM3_ESM.docx]

| Phylum | Family | Altitude (m) ^1^ | | | SEM ^2^ | *P* value |
| --- | --- | --- | --- | --- | --- | --- |
|  |  | L | M | H |  |  |
| *Actinobacteria* | *Coriobacteriaceae* | 2.90^b^ | 4.62^a^ | 2.04^b^ | 0.2539 | < 0.0001 |
| *Bacteroidetes* | *Bacteroidales_UCG-001* | 1.14 | 1.47 | 1.47 | 0.3634 | 0.1434 |
|  | *Bacteroidales_BS11_gut_group* | 8.47 | 9.32 | 8.43 | 0.2005 | 0.1229 |
|  | *Rikenellaceae* | 7.70^b^ | 8.49^a^ | 6.98^c^ | 0.1525 | < 0.0001 |
|  | *Prevotellaceae* | 19.23^a^ | 14.16^b^ | 11.73^c^ | 0.7287 | 0.0047 |
| *Firmicutes* | *Lachnospiraceae* | 9.25 | 10.84 | 10.02 | 0.4160 | 0.0831 |
|  | *Acidaminococcaceae* | 1.65 | 1.93 | 1.83 | 0.2576 | 0.3285 |
|  | *Christensenellaceae* | 11.19^c^ | 14.26^b^ | 15.63^a^ | 0.3905 | < 0.0001 |
|  | *Ruminococcaceae* | 17.12^b^ | 18.76^b^ | 20.70^a^ | 0.3101 | 0.0007 |
|  | *Veillonellaceae* | 2.17 | 2.06 | 2.38 | 0.4267 | 0.1536 |

^a,b,c^ Values in the same row with different superscript letters differ significantly (*P* < 0.05)

^1^ L, 2,800 m; M, 3,700 m; H, 4,700 m

^2^ Standard error of the mean
