# Supplementary material for: Altitude influences microbial diversity and herbage fermentation in the rumen of yaks
Source: BMC Microbiol. 2020 Dec 4;20:370. doi: 10.1186/s12866-020-02054-5 (PMC7718673; doi:10.1186/s12866-020-02054-5)
Supplement: Supplementary file 4 — Additional file 4: Table S4. Comparison of the dominant genera (average relative abundance ≥0.1% for at least one altitude) within the rumen [file 12866_2020_2054_MOESM4_ESM.docx]

| Phylum | Genus | Altitude ^1^ | | | SEM ^2^ | *P* value |
| --- | --- | --- | --- | --- | --- | --- |
|  |  | L | M | H |  |  |
| *Bacteroidetes* | *Prevotella_1* | 13.57^a^ | 10.47^b^ | 10.68^b^ | 0.5314 | 0.0242 |
|  | *Rikenellaceae_RC9_gut_group* | 7.34^b^ | 7.93^a^ | 5.23^c^ | 0.2203 | < 0.0001 |
|  | *Prevotellaceae_UCG-003* | 1.53 | 1.29 | 1.52 | 0.0616 | 0.2057 |
|  | *SP3-e08* | 0.41 | 0.39 | 0.58 | 0.3163 | 0.1831 |
|  | *Prevotellaceae_UCG-001* | 1.85^a^ | 1.59^b^ | 1.20^c^ | 0.1098 | 0.0413 |
|  | *Alloprevotella* | 0.13^b^ | 0.14^b^ | 0.17^a^ | 0.0023 | 0.0122 |
| *Firmicutes* | *Christensenellaceae_R-7_group* | 8.76^b^ | 10.09^a^ | 10.76^a^ | 0.2411 | 0.0011 |
|  | *Ruminococcaceae_NK4A214_group* | 5.41^b^ | 4.37^c^ | 7.72^a^ | 0.2595 | < 0.0001 |
|  | *Succiniclasticum* | 3.83^a^ | 2.93^b^ | 0.65^c^ | 0.2582 | < 0.0001 |
|  | *Butyrivibrio_2* | 3.79^a^ | 1.78^b^ | 0.53^c^ | 0.2379 | < 0.0001 |
|  | *E. coprostanoligenes* | 2.07^b^ | 2.21^ab^ | 2.38^a^ | 0.0493 | 0.0276 |
|  | *Ruminococcaceae_UCG-005* | 1.12 | 1.33 | 1.04 | 0.2721 | 0.0631 |
|  | *Ruminococcaceae_UCG-010* | 1.65 | 1.80 | 1.87 | 0.4565 | 0.3027 |
|  | *Veillonellaceae_UCG-001* | 1.71^b^ | 1.30^c^ | 2.46^a^ | 0.1102 | < 0.0001 |
|  | *Lachnospiraceae_XPB1014_group* | 1.34^a^ | 1.08^b^ | 0.25^c^ | 0.0839 | < 0.0001 |
|  | *Oribacterium* | 0.20^b^ | 1.49^a^ | 0.24^b^ | 0.0243 | 0.0125 |
|  | *Saccharofermentans* | 1.40^b^ | 1.86^a^ | 0.93^c^ | 0.0712 | < 0.0001 |
|  | *Papillibacter* | 0.86 | 0.64 | 0.61 | 0.1362 | 0.0631 |
|  | *Ruminococcaceae_UCG-014* | 0.54^b^ | 1.08^a^ | 0.58^b^ | 0.0445 | < 0.0001 |
|  | *Ruminococcus_1* | 0.40^c^ | 0.70^b^ | 1.18^a^ | 0.0598 | 0.0413 |
|  | *Streptococcus* | 0.11^c^ | 0.13^b^ | 0.54^a^ | 0.0104 | < 0.0001 |
|  | *Clostridium* | 0.03^c^ | 0.14^b^ | 0.31^a^ | 0.0193 | < 0.0001 |
|  | *Romboutsia* | 0.06^c^ | 0.11^b^ | 0.14^a^ | 0.0032 | < 0.0001 |
| *Synergistetes* | *Fretibacterium* | 0.66 | 0.57 | 0.65 | 0.0116 | 0.0516 |
| *Spirochaetae* | *Treponema* | 0.53^b^ | 0.59^ab^ | 0.71^a^ | 0.0327 | 0.0415 |

^a,b,c^ Values in the same row with different superscript letters differ significantly (*P* < 0.05)

^1^ L, 2,800 m; M, 3,700 m; H, 4,700 m

^2^ Standard error of the mean
