# Supplementary material for: Altitude influences microbial diversity and herbage fermentation in the rumen of yaks
Source: BMC Microbiol. 2020 Dec 4;20:370. doi: 10.1186/s12866-020-02054-5 (PMC7718673; doi:10.1186/s12866-020-02054-5)
Supplement: Supplementary file 5 — Additional file 5: Table S5. Major species and proportion of edible herbage at different altitudes [file 12866_2020_2054_MOESM5_ESM.docx]

| Altitude^1^ | Main edible herbage species | Above-ground biomass/(g/m^2^) | Edible herbage/(g/m^2^) | Inedible herbage/(g/m^2^) |
| --- | --- | --- | --- | --- |
| L | Sedges: *Kobresia capillifolia*, *Scirpus pumilus*; | 374 | 208 | 166 |
|  | Grasses: *Elymus nutans*, *Poa pratensis*; |  |  |  |
|  | Forbs: *Saussurea hieracioides*, *Halenia corniculate*, *Anaphalis lacteal*, [*Potentilla anserina*](http://frps.iplant.cn/frps/Potentilla%20anserina); |  |  |  |
|  | Shrubs: *Potentilla fruticose* |  |  |  |
| M | Sedges: *Kobresia capillifolia*, *Carex thibetica*; | 325 | 167 | 158 |
|  | Grasses: *Elymus nutans*, *Poa pratensis*, *Stipa aliena*; |  |  |  |
|  | Forbs: *Anemone rivularis var*. *flore-minore*, *Anemone trullifolia var*. *linearis*, *Pedicularis chinensis*, *Halenia corniculate*, *Ligularia virgaurea*, *Saussurea hieracioides*, *Anaphalis lacteal*, [*Potentilla anserina*](http://frps.iplant.cn/frps/Potentilla%20anserina); |  |  |  |
|  | Shrubs: *Artemisia mongolica*, *Potentilla fruticose* |  |  |  |
| H | Sedges: *Kobresia humilis*, *Kobresia tibetica*, *Kobresia pygmaea*, *Carex moorcroftii*; | 280 | 158 | 122 |
|  | Grasses: *Stipa purpurea*; |  |  |  |
|  | Forbs: *Leontopodium nanum*, *Artemisia frigida willd*, *Polygonum macrophyllum*, *Potentilla saundersiana*; |  |  |  |
|  | Shrubs: *Salix xizangensis*, *Potentilla fruticose* |  |  |  |

L, 2,800 m; M, 3,700 m; H, 4,700 m
